# The Effect of the Layered Internal Structure of Fibrous Beds on the Hydrodynamic Diffusive Behavior of Microparticles

**DOI:** 10.3390/mi12101241

**Published:** 2021-10-13

**Authors:** Ryoko Otomo, Ryosuke Kira

**Affiliations:** Faculty of Engineering Science, Department of Mechanical Engineering, Kansai University, 3-3-35 Yamate-cho, Suita, Osaka 564-8680, Japan; k181590@kansai-u.ac.jp

**Keywords:** fibrous bed, stokesian dynamics, microparticle, hydrodynamic interaction

## Abstract

To separate and collect microparticles such as cells, the behavior of particles in fibrous filters was investigated. It is essential to understand, in detail, the motion of particles in microscale flows, because *Re* is often small, and particles exhibit complex behaviors such as changes in relative position and spreading owing to hydrodynamic interactions. We calculated the motion of microparticles passing through the fibrous bed using the Stokesian dynamics method, in which hydrodynamic interaction is considered, theoretically. The fibrous bed was modeled by particles and five types of structures (a monolayer with fiber volume fractions *φ* of 3%, 4%, and 5%, and a bilayer with *φ* = 3%−5% and 5%−3%) were considered. Our numerical results showed that the particles moved in a complicated manner, and spread throughout the fibrous bed. It was found that the behavior of individual microparticles varied depending on the internal structure, although the average permeation velocity was primarily determined by the fiber volume fraction. This great dependence of the behavior of particle assemblage on the internal structure of the fibrous bed was caused by the individual particle motion under the influence of the layers in front of and behind them, owing to the hydrodynamic interaction.

## 1. Introduction

In recent years, increasing attention has been paid to technologies for collecting, separating, and manipulating cells and microparticles. One of the most interesting fields is microfluidics, in which particles are manipulated non-invasively by using channels with a fine microstructure. This method takes advantage of the characteristics of flow fields or fine particles, such as the existence of secondary flow, inertial migration, and the optical characteristics of the particles. A number of studies have been performed to discover novel separation methods and develop suitable channel shapes [1,2]. However, in microfluidics, many techniques are required to manufacture microchannels with a fine structure, handle fine particles, and allow fine particle suspensions to flow properly in the channel.

Filtration by microfibers can be a simpler method for separating and collecting fine particles such as cells. For example, a high-efficiency particulate air filter, which has a high collection efficiency, is used to remove microparticles suspended in air or liquid. This can be used to provide a clean air environment or wastewater treatment for places such as precision equipment manufacturing sites, food factories, and medical research facilities, which require accurate particle separation [3].

Another example of cell separation using fibrous filters is the platelet-rich plasma (PRP) filter, which separates white blood cells from platelets in the blood to prepare a PRP solution [4]. Platelets have a healing function that repairs damaged tissues and are used to treat injuries. The PRP solution is injected into the injured cells to accelerate healing. It is also used for injuries that are normally difficult to heal [5,6]. The ideal PRP filter completely removes white blood cells and allows all necessary platelets to permeate. However, a certain number of platelets are removed along with the white blood cells. In previous studies, the most optimized conditions that facilitate the collection of white blood cells and minimize the number of platelets removed have been discussed by examining the effects of fiber volume fraction, fiber diameter, fibrous filter thickness, and flow rate for injecting cells [4].

In addition to collection and separation, it is also important to understand in vivo transport phenomena, such as the transport of substances in collagen gels. For tumor treatment, therapeutic drugs are delivered to the target cancer cells. The drugs are transported via the extracellular matrix, which is a fibrous medium composed of proteins, including collagen. Therefore, the transport behavior of drugs in fibrous collagen gels determines access to cancer cells [7,8]. In previous studies, the diffusivity of spherical macromolecules in collagen gel was evaluated using the orientation of fibers and the size of fine particles as parameters [8,9].

From the above examples, it is important to understand the collection, separation, and transport phenomena of microparticles in fibrous media. In previous studies, macroscopic transport characteristics, such as the collection efficiency and diffusion coefficient of particles, have often been investigated. However, for more efficient and effective applications, a fundamental understanding of how each particle behaves in the fibrous bed under various conditions, such as the particle size and the internal structure of the bed, is also important.

The motion of microparticles in a fluid is often under low Reynolds number conditions. Because the fluid viscosity is dominant (*Re* → 0), the hydrodynamic interaction among particles via the fluid greatly affects the behavior of the particles. It has been reported that when an assemblage of microparticles moves in a liquid, it shows a spreading behavior because the motion of individual particles is affected by the flow field under the influence of the other particles [10,11,12]. This phenomenon is called hydrodynamic diffusion, which is different from the molecular diffusion caused by thermal motion (Brownian motion). Furthermore, in the fibrous bed, hydrodynamic interaction occurs between particles and fibers, as well as between particles. Because hydrodynamic interactions range over long-distances, the motion of one particle is determined by fibers and other particles, not only in the vicinity but also far in the distance. Therefore, the internal structure of the fibrous bed and the size of particles greatly affect the motion of the entire particle assemblage and, consequently, such motion becomes extremely complicated.

Regarding the effect of fibers on the flow, many studies have evaluated fluid permeability in fibrous media [13,14,15,16]. In such studies, straight fibers have often been modeled as cylinders, and the effects of fiber volume fraction and fiber orientation have been evaluated. To express a fibrous bed with complicated internal structures that cannot be modeled as cylinders, in a previous study we modeled the tortuous fibers as static particles, and simulated the behavior of microparticles moving around them. As a result, it was shown that the behavior of individual particles depends on the shape (tortuosity) of the fiber, and that the fiber shape has a significant influence on the hydrodynamic diffusion of entire particles [17]. However, actual fibrous materials often have more complicated internal structures. For example, dual layered fibrous filters, where layers with different functions and purposes are combined, are used for air filtration of nanoparticles [18]. The effects of layered structures on the hydrodynamic motion of particles through the fibrous bed were not adequately revealed.

In the present study, we numerically simulated the motion of microparticles through a fibrous bed, where the pores were saturated by a fluid. Similar to Otomo and Mori [17], the fibrous bed was expressed by particles, and the behavior of the microparticles was calculated using the Stokesian dynamics method [19,20,21], assuming Stokes flow. According to the permeation of platelets in a PRP filter, the size of the microparticles was set to one to two times the fiber radius. As the internal structure, we used a monolayer structure (in which the fiber volume fraction in the bed was constant) and a bilayer structure (consisting of layers with two different volume fractions). Based on the difference in the behavior of particles in these fibrous beds, the effects of the layered internal structure of the fibrous bed on the hydrodynamic diffusion of the particle assemblage were investigated.

## 2. Calculation Methods

### 2.1. Stokesian Dynamics

In this study, we assumed *Re* << 1 and *St* << 1 and calculated the behavior of microparticles in the Stokes flow field using the Stokesian dynamics (SD) method [19,20,21]. SD describes the relative motion between particles and a fluid by considering hydrodynamic interactions in the presence of multiple particles. Based on the Stokes equation and the equation of continuity of an incompressible fluid, the flow field caused by the existence of *N* particles was derived using multipole expansion as follows:

(1) $$v_{i}\left(x\right)=u_{i}^{\infty }\left(x\right)+\frac{1}{8\pi \mu }\overset{N}{\underset{\beta =1}{\sum }}\left[\left(1+\frac{1}{6}a^{2}\nabla^{2}\right)J_{ij}\left(r\right)F_{j}^{\beta }+R_{ij}\left(r\right)T_{j}^{\beta }-K_{ijk}\left(r\right)S_{jk}^{\beta }\cdots \right]$$

where *μ* is the fluid viscosity and ***u***^∞^ is the flow field without particles. ***F****^β^*, ***T****^β^*, and ***S****^β^* are the force, torque, and stresslet exerted on the flow by particle *β*, respectively. ***J***(***r***), ***R***(***r***), and ***K***(***r***) are expressed as follows:

(2) $$J_{ij}\left(r\right)=\frac{\delta_{ij}}{r}+\frac{r_{i}r_{j}}{r^{3}},\;R_{ij}\left(r\right)=\varepsilon_{ijk}\frac{r_{k}}{r^{3}},\;K_{ijk}\left(r\right)=-3\frac{r_{i}r_{j}r_{k}}{r^{5}}$$

The value ***r*** represents ***r*** = ***x*** − ***x****_β_*, where ***x****_β_* is the center of particle *β*. Because the particle volume fraction is small, we used the F version of the SD method, in which only the translational motion of particles is considered [4,22]. From Faxèn’s law and Equation (1), in which the high-order terms are omitted because of the F version, the relationship between the relative velocity between the particles and the fluid, ***U*** − ***u***^∞^, and the hydrodynamic force ***F*** exerted by each particle on the fluid is expressed as follows [23]:

(3) $$U-u^{\infty }=M\cdot F$$

***M*** is called the mobility matrix (or the Rotne−Prager tensor, particularly for the F version), which is determined by the positional relationship and size of all particles. To consider the near-field (lubrication) effects between particles, ***M*** in Equation (3) was corrected based on the pairwise interaction [24]. Force ***F*** has an action−reaction relationship with the fluid force ***F*_f_** acting on each particle. When the particle inertia is negligible, the total force acting on the particle, fluid force, repulsive force ***F*_rep_** explained in Section 2.2, and external force ***F*_ext_** explained in Section 2.3, are balanced.

In the case where there are two types of particles—static particles (fiber particles) fixed in a fluid and the microparticles moving around them—the velocity of the moving microparticles is obtained by the following equation, which is derived from Equation (3) [25,26]:

(4) $$U^{m}=u^{\infty }+{\left(R^{mm}\right)}^{-1}\cdot \left(F-R^{ms}\cdot u^{\infty }\right)$$

In Equation (4), the superscripts *s* and *m* represent the fiber (static) particles and microparticles, respectively. ***R*** is the part extracted from the inverse matrix of ***M***, which expresses the interaction between each type of particle.

The particle positions are calculated by the fourth-order Runge−Kutta method using the velocities ***U****^m^* of each particle obtained by Equation (4). All calculations were performed in a dimensionless manner using the representative quantities shown in Section 2.3. The validity of the calculation of particle behavior using the SD method (F version), and the validity of the representation of fibers using particles shown in Section 2.3, have been confirmed in previous studies by the authors [17].

### 2.2. Repulsive Force Acting on Microparticles and Fiber Particles

To prevent the overlapping of particles, the following non-dimensional repulsive forces were applied between all fiber particles and microparticles, and between microparticles, according to Nott and Brady [25,26]:

(5) $$F_{rep}\left(D\right)/6\pi \mu a_{m}U=F_{0}\frac{\tau \exp\left(-\tau D/a\right)}{1-\exp\left(-\tau D/a\right)}e$$

where *D* is the distance between the particle surfaces, and ***e*** is the unit vector of the direction in which two particles repel each other. In this study, non-dimensional parameters were set to *F*_0_ = 0.001 and *τ* = 1000 so that the repulsive force acts only when the particles are quite close to each other. Therefore, this repulsive force had a negligible effect on the results of the particle behavior. These values were determined according to Nott and Brady [25].

### 2.3. Calculation Conditions

**Figure 1:**
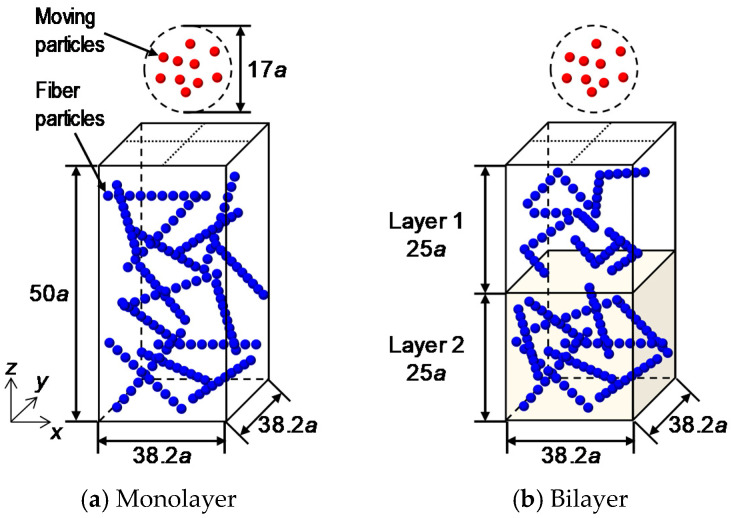
Calculation domain.

Figure 1 shows the computational domain. In this study, a single fiber was represented by an array of 10 fiber particles with an interparticle distance of 0.1*a* (*a*: fiber radius), and the fibrous bed region was set to 38.2*a* × 38.2*a* × 50*a*. The number of particles per single fiber and the interparticle distance were confirmed to have no qualitative influence on the flow characteristics in the bed. The pores and the outside of the fibrous bed region were filled with fluid, that is, the periodic boundary condition was not applied to the calculation by the SD method. The fibers were arranged in random positions and orientations to satisfy the fiber volume fraction *ϕ*_f_, as shown in Table 1. In the case of the bilayer structure, the fibrous bed region was divided into Layer 1 and Layer 2 at *z* = 25*a*, where the particles moved in the *z*-direction. The volume fraction was expressed as *ϕ*_f_ = 3%−5%, for example, if the fiber volume fraction of Layer 1 was 3% and that of Layer 2 was 5%. If a fiber was located outside of the fibrous bed region or each layer (in the bilayer cases), the position of the outside particles was modified in a periodic boundary manner. We performed six calculations with different initial arrangements of the fibers and microparticles for each condition.

The material moving through the fibrous bed was represented by 20 microparticles of radius *a*_m_. An external force ***F*_ext_**/6*πμa*_m_*U* = (0, 0, −1) was applied to each microparticle in a static fluid (***u***^∞^ = **0**). The initial positions of the microparticles were randomly placed within the range of a sphere of radius 8.5*a*, whose center coordinates were (19.1*a*, 19.1*a*, 60.5*a*) in the upper part of the fibrous bed region, without overlapping. The particle radius was set to two conditions: *a*_m_ = *a*, which was equal to the fiber radius, and *a*_m_ = 2*a*, which was twice the fiber radius. To prevent the overlapping of particles, repulsive forces between the particles described in Section 2.2 were considered.

The representative amount of non-dimensionalization was determined based on platelet permeation in the PRP filter. Fluid viscosity *μ* = 1.0 mPa∙s, representative length (fiber radius) *a* = 1.5 μm, and representative velocity *U* = 300 μm/s [4] were used. In this study, where the fiber and microparticle volume fraction was loose, the particles did not get too close to each other. The rapid change of the velocity of particles did not occur, except for some rare cases. The dimensionless time increment for calculating the particle behavior was set to Δ*t*/(*a*/*U*) = 0.05, which was small enough to avoid the overlapping caused by the infrequent approach of particles.

## 3. Results

### 3.1. Permeation and Capture of Microparticles through Fibrous Bed

**Figure 2:**
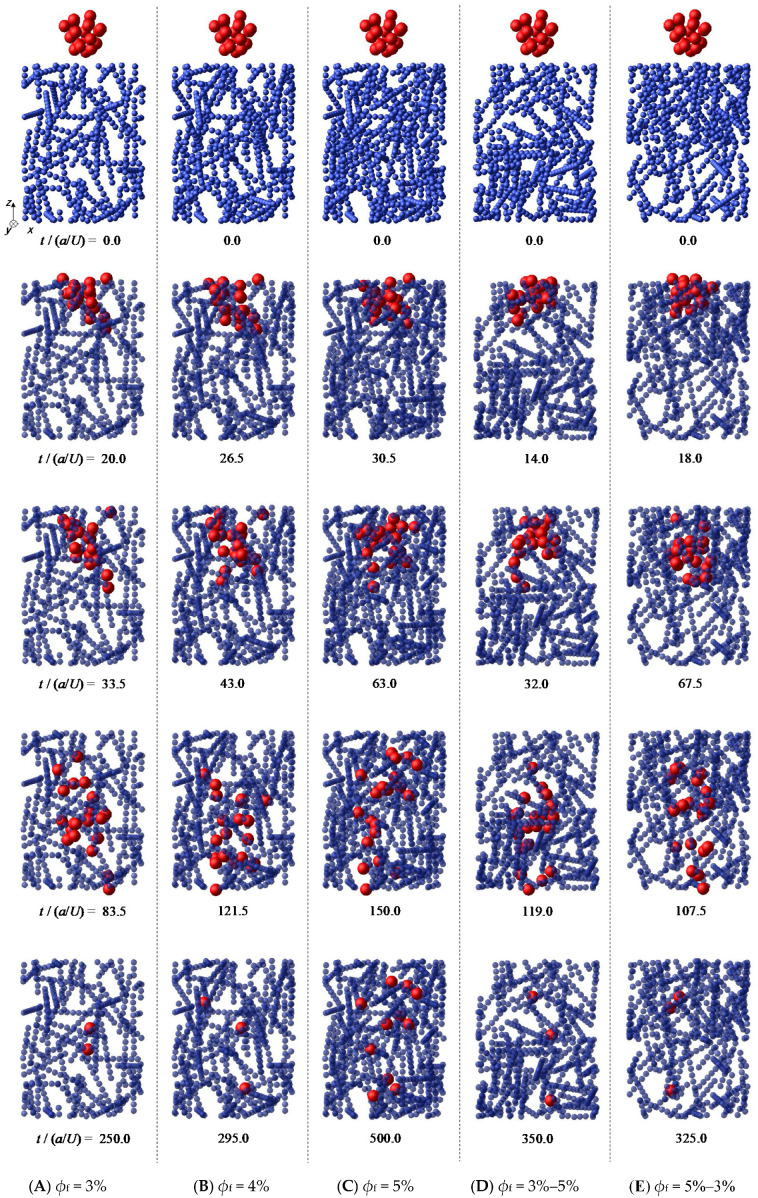
Permeation behavior of microparticles through fibrous beds (*a*_m_ = 2*a*).

As an example of the simulation results, the behavior of microparticles for *a*_m_ = 2*a* is shown in Figure 2. The results indicate that each particle moved in a complicated manner, avoiding fibers and other particles. This motion is caused by the hydrodynamic interactions and is characteristic of Stokes flow, as is often the case in microscale phenomena. As a result of this complex behavior, under all conditions, the microparticles spread while passing through the fibrous bed, which is interpreted as hydrodynamic diffusion.

**Figure 3:**
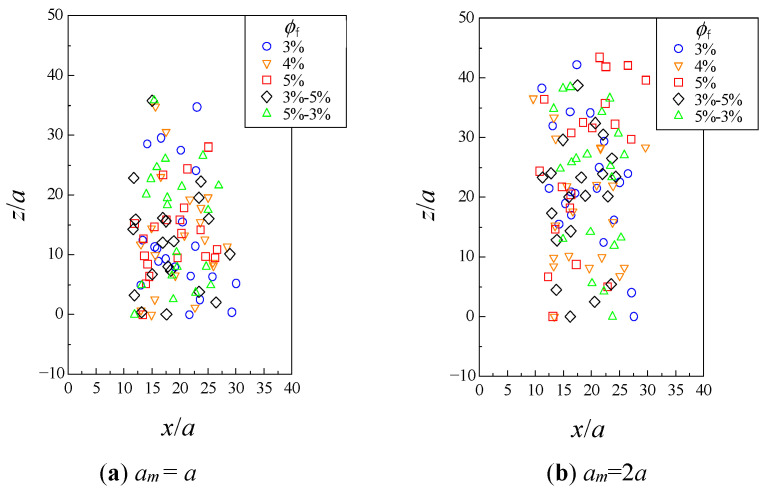
Distribution of particles in fibrous bed from *x*−*z* plane.

**Figure 4:**
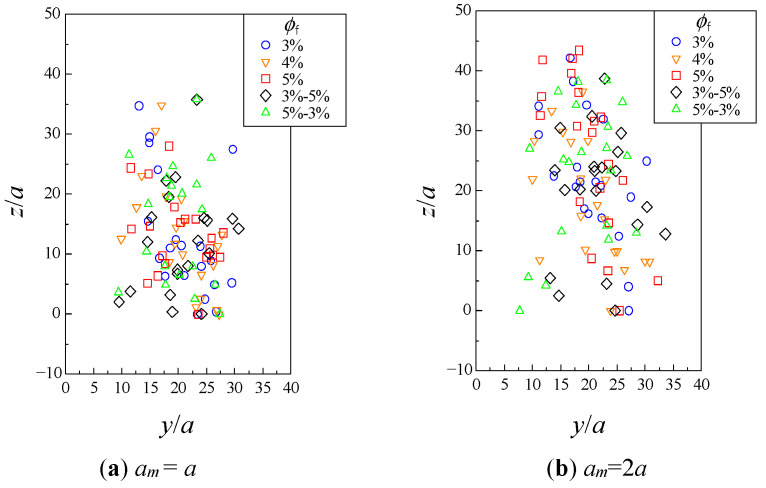
Distribution of particles in fibrous bed from *y*−*z* plane.

Figure 3 and Figure 4 show the distribution of the particles viewed from the *x*−*z* and *y*−*z* planes, at the time when the leading particle exited the fibrous bed. The fibrous bed was located at *z*/*a* = 0−50. These results were obtained from one of the six trials with different fiber configurations and initial particle configurations. When *a*_m_ = *a*, there was no significant difference owing to the fibrous bed structure. When *a*_m_ = 2*a*, it was noticeable that there were some particles that did not progress after entering the fibrous bed under the conditions *ϕ*_f_ = 5% and *ϕ*_f_ = 5%−3%.

As shown in Figure 2, some particles were stuck because they were blocked by the fiber particles. These were considered to be "captured" particles. Table 2 shows the number of particles captured in Layer 1, Layer 2, and the total, averaged over the six trials. Table 2 suggests that, in the case of the monolayer structure, the larger the fiber volume fraction, the higher the number of particles captured, regardless of the particle size. This is because the fibrous bed with the larger volume fraction included smaller pores through which the particles could pass. On the other hand, comparing the results of *ϕ*_f_ = 5%, that is, the total of the monolayer with *ϕ*_f_ = 5%, Layer 2 with *ϕ*_f_ = 3%−5%, and Layer 1 with *ϕ*_f_ = 5%−3%, we found that the number of captured particles was different, even though the fiber volume fraction was the same. Furthermore, the number of captured particles was also different in the fibrous bed with *ϕ*_f_ = 4%, *ϕ*_f_ = 3%−5%, and *ϕ*_f_ = 5%−3%, although the fiber volume fraction of the whole bed was the same. This difference in the number of captured particles is thought to be caused by the behavior of the particles inside the fibrous bed. The behavior of the particles that were not captured was investigated in detail and is discussed in the following sections.

### 3.2. Hydrodynamic Diffusion of Microparticles

#### 3.2.1. Permeation Time and Distance Traveled by Particles

The distance traveled by each particle in the *z*-direction between time *t*_0_ (when the leading particle of the assemblage entered the fibrous bed) and time *t*_1_ (when it exited the bed) was examined. The average distance traveled by each particle, *δ*_z_, and its variation, *σ*_δz_, were calculated.

(6) $$\langle \delta_{z}\left(t\right)\rangle =\frac{1}{n_{m}}\overset{n_{m}}{\underset{i=1}{\sum }}\int_{t_{0}}^{t}v_{z,i}\left(t'\right)dt'=\frac{1}{n_{m}}\overset{n_{m}}{\underset{i=1}{\sum }}\delta_{z,i}\left(t\right)$$

(7) $$\sigma_{\delta z}\left(t\right)=\sqrt{\frac{\sum_{i=1}^{n_{m}}{\left\{\delta_{z,i}\left(t\right)-\langle \delta_{z}\left(t\right)\rangle \right\}}^{2}}{n_{m}}}$$

**Figure 5:**
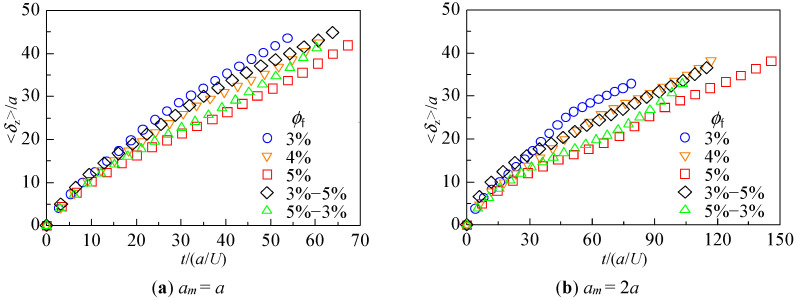
Average distance traveled by 20 particles.

**Figure 6:**
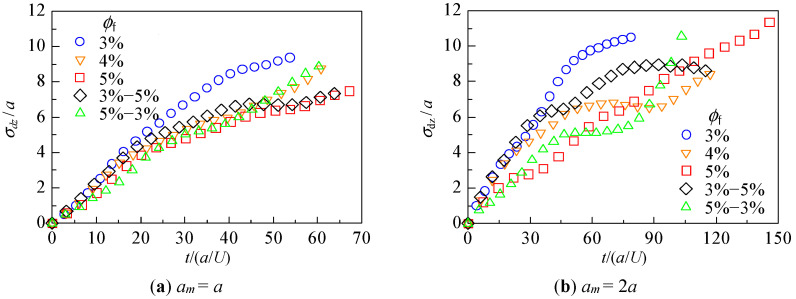
Variation of distance traveled by 20 particles.

Here, *v*_z,*i*_(*t*) is the *z*-component of the velocity of particle *i* at time *t* and *n_m_* is the number of particles, excluding those that were captured. A large <*δ*_z_> indicates that many particles traveled a longer distance, whereas a large *σ*_δz_ indicates that there was a difference in the distance traveled by individual particles. The results are shown in Figure 5 and Figure 6, respectively. The horizontal axis is the dimensionless time *t*/(*a*/*U*) with *t*_0_ = 0, and *t*_1_ is indicated by the end of the plot for each condition.

As shown in Figure 5, the average distance <*δ*_z_> in time *t*_1_ for each particle size condition was not significantly different, regardless of the fibrous bed structure. The time *t*_1_ taken for the leading particle to pass through the fibrous bed was longer for a larger fiber volume fraction in the case of the monolayer structure. The results for *ϕ*_f_ = 4%, 3%−5%, and 5%−3% also showed that *t*_1_ was similar for equal volume fractions of the entire bed, despite different internal structures.

Figure 6 shows that when *a_m_* = *a*, *σ*_δz_ was larger for the fiber volume fraction in the order of 3% = 5%−3% = 4% > 5%−3% = 5%, and when *a_m_* = 2*a*, *σ*_δz_ was larger in the order of 5% > 3% = 5%−3% > 4% = 3%−5%. For the bilayer structure, a large difference was observed, especially in the latter behavior. In Figure 6a,b, the slope of *σ*_δz_ increases sharply for *ϕ*_f_ = 3%−5% (green triangles). The increased difference in the distance traveled by each particle would result in a large overall spread of the moving assemblage. On the contrary, the slope decreases over time for *ϕ*_f_ = 5%−3% (black diamonds), indicating that the traveled distance becomes similar among particles, that is, the spreading of the particles is suppressed.

#### 3.2.2. Velocity in the Travel Direction

**Figure 7:**
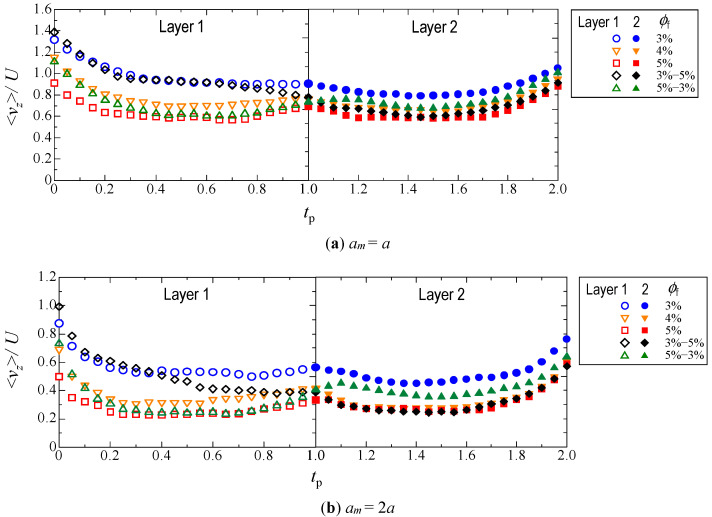
Velocity in the *z*-direction which is averaged over 20 microparticles.

Figure 7 shows the velocity in the *z*-direction <*v*_z_>, which was averaged over 20 microparticles. Individual particles passed through the Layer 1 entrance, the Layer 2 entrance, and the Layer 2 exit at different times. We plotted the velocity of each particle with respect to the normalized time *t*_p_, which took the value of *t*_p_ = 0 when the particle entered Layer 1, *t*_p_ = 1 when it entered Layer 2, and *t*_p_ = 2 when it exited Layer 2. Finally, the velocities of all particles at the same *t*_p_ were averaged. 

For the monolayer structure, the larger the fiber volume fraction, the slower the *z*-direction velocity. For the bilayer structure, Layer 1 with a smaller volume fraction had a higher velocity than Layer 2 when *ϕ*_f_ = 3%−5% (black diamonds), whereas Layer 1 with a larger volume fraction had a slower velocity than Layer 2 when *ϕ*_f_ = 5%−3% (green triangles). This is consistent with the results shown in Figure 5 and Figure 6, thus confirming that the average moving speed of the particle assemblage was determined by the fiber volume fraction. Therefore, in the bilayer structure, the resistance during movement was different in each layer.

A more detailed observation of the change in velocity showed that the velocity varied, even when the particles passed through the part where the volume fraction was uniform. For example, in Layer 1 with *ϕ*_f_ = 3%−5% (hollow black diamonds), it was observed that the velocity gradually decreased as the particle approached Layer 2 (*t*_p_ = 1). This was owing to the large volume fraction in Layer 2, which suggests that the particles were still in Layer 1 but were affected by fibers in Layer 2. In contrast, Layer 1 with *ϕ*_f_ = 5%−3% (hollow green triangles) showed a slight increase in velocity just before *t*_p_ reached unity, owing to the small volume fraction in Layer 2. A similar trend was observed at the end of Layer 2. Owing to the finite thickness of the fibrous bed, and the absence of fibers after Layer 2, the velocity (solid symbols) for all conditions increased as *t*_p_ approached 2.

#### 3.2.3. Change in Dispersion of Particles Due to Hydrodynamic Behavior

**Figure 8:**
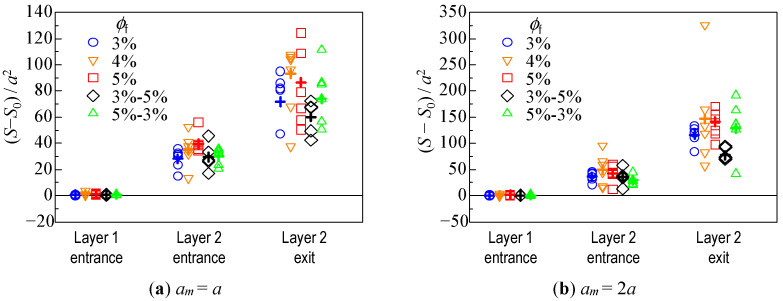
Dispersion of the position of each particle with respect to the center of the assemblage.

**Figure 9:**
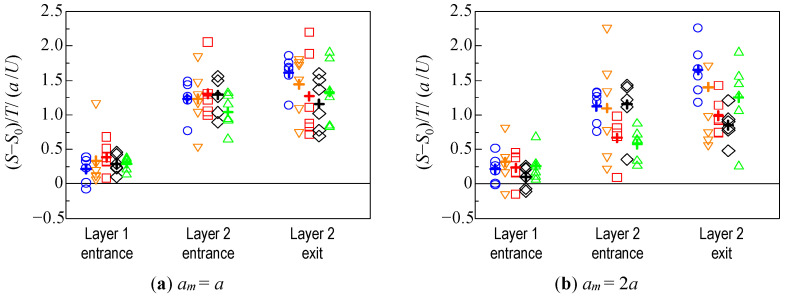
Dispersion of the position of each particle based on permeation time. The legend symbols are the same as Figure 8.

As a quantitative indicator of the spreading of the particle assemblage, the dispersion of the position of each particle, with respect to the center of the assemblage, was investigated.

(8) $$S\left(t\right)=\frac{\sum_{i=1}^{n_{m}}{\left\{z_{i}\left(t\right)-\langle z\left(t\right)\rangle \right\}}^{2}}{n_{m}}$$

where <*z*(*t*)> is the position of the center of mass of the particle assemblage at time *t*. The variance *S*_0_ of the position at the initial state was used as a reference, and the variance at the time when the leading particle reached the entrance of Layer 1, the entrance of Layer 2, and the exit of Layer 2, was examined. The results of the six trials are shown in Figure 8. Because the time taken to pass through the fibrous bed was different depending on the condition, as shown in Figure 5 and Figure 6, Figure 9 shows the variance of the position $\left(S-S_{0}\right)/T$, which takes this into account. *T* is the time taken for the leading particle to pass through each layer. In Figure 8 and Figure 9, the average of the results under each condition are shown by crosses.

Figure 8 shows that, for the monolayer structure, when the fiber volume fraction was larger, the dispersion of the particles was almost greater as they passed through the bed. The results of *ϕ*_f_ = 4% for the monolayer and bilayer structures suggest that the spreading tendency of the microparticles was significantly different, despite the same volume fraction of the entire bed. For *ϕ*_f_ = 5%−3%, the dispersion did not change significantly up to the entrance of Layer 2, whereas the increase in dispersion through Layer 2 was very large. On the contrary, for *ϕ*_f_ = 3%−5%, the increase in dispersion was relatively small throughout the fiber layer, indicating that the particle assemblage did not spread much.

From Figure 9, it was found that the smaller the fiber volume fraction, the larger the positional dispersion rate in the monolayer structure when the particle reached the Layer 2 exit. This is different from the trend shown in Figure 8, although there is some variation owing to the initial position of the particles. In the case of the bilayer structure, the spreading behavior of the particles was similar to that shown in Figure 8, regardless of the permeation time.

## 4. Discussion

When the approximations *Re* → 0 and *St* → 0 hold, the lubrication effect prevents collisions between particles, and the particles move in a complicated manner while changing their positions relative to each other. In the present results, the collision did not occur, and the motion of particles, affected by their positional relationship with other particles and fibers, can be reproduced. As a consequence of this complex behavior, it was observed in Figure 2 that the entire particle assemblage spread throughout the fibrous bed. In the author’s previous study, it was reported that hydrodynamic diffusion through an obstacle such as a fibrous bed was more pronounced than when the particles simply moved in a liquid [17]. The present results also show that some particles were hindered by the fibers and others were not, which might have led to the large spreading. From Figure 3 and Figure 4 and Table 2, it can be seen that the position and number of particles collected varied depending on the fibrous bed structure, indicating that the structure had some effect on the behavior of individual particles.

Figure 5 shows that the permeation time (the time taken for the leading particle to pass through the entire fibrous bed) and the average travel distance depended more on the fiber volume fraction than on the internal structure of the bed. Although the behavior of individual particles varied depending on the internal structure, as reported in Section 3.1, Figure 5 indicates that the average velocity of particle assemblage was primarily determined by the fiber volume fraction. In contrast, Figure 6 shows that the spreading of the particle assemblage was greatly affected by the internal structure of the fibrous bed. We can see from Figure 2 that for both *ϕ*_f_ = 3%−5% and *ϕ*_f_ = 5%−3%, many particles remained in Layer 1 when the leading particles entered Layer 2. In the case of *ϕ*_f_ = 5%−3%, the rear particles in Layer 1, which had a large fiber volume fraction, were difficult to move, and their *z*-direction velocity may have been slower than that of the leading particles moving in Layer 2. Therefore, when the volume fraction differs within the fibrous bed, a velocity difference is generated between particles, resulting in behavior where the difference between the backward and leading particles increases rapidly, or where the backward particles catch up with the leading particles. Thus, even for the same combination of fiber volume fractions, the behavior of the particles differs greatly depending on the positional relationship of the layers.

The particle velocity through Layers 1 and 2 (Figure 7) indicates that the average velocity is determined by the fiber volume fraction, which is consistent with the discussion for Figure 5 and Figure 6. This suggests that the combination of two layers with different volume fractions results in different particle velocities in each layer, which determined the spreading behavior of the particle assemblage shown in Figure 6. Furthermore, as shown in Figure 7, the velocity is not time-homogeneous; instead, it changes even within a single layer. In particular, in the case of the bilayer structure, the average velocity of Layer 1 was determined by the volume fraction of Layer 1, but it was also affected by the volume fraction of Layer 2. This is because of the long-range effect, which is a characteristic of Stokes flow where the influence of viscosity is relatively large. Therefore, it is clear that the particle velocity is affected not only by the layer in which the particles are located at the time, but also by the layers in front of and behind them. Consequently, the fibrous bed structure has a complex effect on the spread of the entire particle assemblage.

Figure 8 and Figure 9 show the quantitative evaluation of the effect of the fibrous bed structure on the spreading of the particles. In the case of a monolayer structure, the particles are likely to spread throughout the fibrous bed with a larger volume fraction, owing to the slower velocity of the particles hindered by the fiber. The spreading rate, which considers the permeation time, is smaller for the larger volume fraction condition, suggesting that the particles pass through the fibrous bed more slowly and with greater spread. 

In the bilayer structure, the spreading behavior of the particle assemblage changes before and after the leading particle enters Layer 2. When the particles move from a large volume fraction layer to a small volume fraction layer, such as *ϕ*_f_ = 5%−3%, a large velocity difference is created between the leading particle and the slower particles in the rear after the leading particle enters Layer 2, resulting in a significant spreading of the particle assemblage (two to three times the difference of *S* between the Layer 2 entrance and exit, in the present case). On the other hand, when moving from a layer with a small volume fraction to one with a large volume fraction, the particle assemblage did not spread as much (about 1.5 times the difference of *S* between the Layer 2 entrance and exit, in the present case) because the leading particle was hindered by the layer with a large volume fraction, and the rear particles caught up with them. Comparing the results for the same volume fraction in Figure 8, the average of *S*–*S*_0_ for 3%−5% was 0.64 times (*a*_m_ = *a*) and 0.53 times (*a*_m_ = 2*a*) that for 4% at the exit of Layer 2. The average of *S*–*S*_0_ for 5%−3% was 0.79 times (*a*_m_ = *a*) and 0.88 times (*a*_m_ = 2*a*) that for 4%, although it was greater than that for 3%−5%. In Figure 9, which takes into account the permeation time, the average of *S*–*S*_0_ /*T* for 3%–5% is 0.80 times (*a*_m_ = *a*) and 0.61 times (*a*_m_ = 2*a*), and that for 5%–3% is 0.92 times (*a*_m_ = *a*) and 0.89 times (*a*_m_ = 2*a*) the average for 4%. It was found that the spreading rate for the bilayer fibrous beds could be nearly half as small as that for the monolayer bed.

To summarize, the behavior of the entire particle assemblage varies greatly depending on the internal structure of the fibrous bed, because the long-range effect of the Stokes flow allows individual particles to move under the influence of the layers in front of and behind them. In the present study, it was not possible to relate the behavior of individual particles to macroscopic properties such as the diffusion coefficient, because the thickness of the fiber layer was not large enough. This will be the focus of our future work. When dealing with a thick fibrous bed, it is expected that the knowledge obtained from the present results can be utilized, because the positional relationship of the particles formed near the bed entrance will affect the subsequent behavior.

## 5. Conclusions

To investigate the effect of a layered internal structure on the hydrodynamic behavior of a microparticle assemblage that passes through a fibrous bed, we calculated the motion of microparticles using the Stokesian dynamics method. Our numerical results reproduced the particle behavior characteristic of the Stokes assumption, where the particles move in a complicated manner and spread throughout the fibrous bed. It was found that the behavior of individual microparticles varied depending on the internal structure, although the average permeation velocity was primarily determined by the fiber volume fraction. This great dependence of the behavior of the particle assemblage on the internal structure of the fibrous bed is caused by the individual particle motion under the influence of the layers in front of and behind them, because of the hydrodynamic interaction. Although there is some variation due to the initial position of the particles, the spreading rate for the bilayer fibrous beds could be nearly half as small as that for monolayer bed, even under the same volume fraction. Relating the behavior of individual particles to macroscopic properties, such as the diffusion coefficient, is part of our planned future work.

## Figures and Tables

**Table 1:** Fiber volume fraction and number of fiber particles.

Adding #	Fiber Volume Fraction *ϕ*_f_		Number of Fiber Particles	
	Layer 1	Layer 2	Layer 1	Layer 2
(A)	3%		520	
(B)	4%		700	
(C)	5%		880	
(D)	3%	5%	260	440
(E)	5%	3%	440	260

**Table 2:** Number of captured particles in layers 1 and 2 of the fibrous bed.

#	*ϕ*_f_	*a*_m_ = *a*			*a*_m_ = 2*a*		
		Layer 1	Layer 2	Total	Layer 1	Layer 2	Total
(A)	3%	0.3	0.0	0.3	1.3	1.0	2.3
(B)	4%	1.3	0.5	1.8	3.0	2.5	5.5
(C)	5%	0.5	0.8	1.3	4.8	3.8	8.6
(D)	3%–5%	0.5	0.3	0.8	1.7	3.5	5.2
(E)	5%–3%	1.2	0.2	1.4	3.5	1.3	4.8
